# MicroRNA-centered theranostics for pulmoprotection in critical COVID-19

**DOI:** 10.1016/j.omtn.2024.102118

**Published:** 2024-01-10

**Authors:** Manel Perez-Pons, Marta Molinero, Iván D. Benítez, María C. García-Hidalgo, Shambhabi Chatterjee, Christian Bär, Jessica González, Antoni Torres, Ferran Barbé, David de Gonzalo-Calvo, CIBERESUCICOVID Project (COV20/00110, ISCIII)

**Affiliations:** 1 Translational Research in Respiratory Medicine, University Hospital Arnau de Vilanova and Santa Maria, IRBLleida, Lleida, Spain; 2 CIBER of Respiratory Diseases (CIBERES), Institute of Health Carlos III, Madrid, Spain; 3 Institute of Molecular and Translational Therapeutic Strategies (IMTTS), Hannover Medical School, Hannover, Germany; 4 Fraunhofer Institute for Toxicology and Experimental Medicine (ITEM), Hannover, Germany; 5 Servei de Pneumologia, Hospital Clinic, Universitat de Barcelona, IDIBAPS, Barcelona, Spain; 6 Institució Catalana de Recerca i Estudis Avançats (ICREA), Barcelona, Spain

**Keywords:** MT: Novel therapeutic targets and biomarker development Special Issue, post-COVID syndrome, long COVID, microRNA, pulmoprotection, theranostics, telomere, telomere length

## Abstract

Elucidating the pathobiological mechanisms underlying post-acute pulmonary sequelae following SARS-CoV-2 infection is essential for early interventions and patient stratification. Here, we investigated the potential of microRNAs (miRNAs) as theranostic agents for pulmoprotection in critical illness survivors. Multicenter study including 172 ICU survivors. Diffusion impairment was defined as a lung-diffusing capacity for carbon monoxide (D_LCO_) <80% within 12 months postdischarge. A disease-associated 16-miRNA panel was quantified in plasma samples collected at ICU admission. Bioinformatic analyses were conducted using KEGG, Reactome, GTEx, and Drug–Gene Interaction databases. The results were validated using an external RNA-seq dataset. A 3-miRNA signature linked to diffusion impairment (miR-27a-3p, miR-93-5p, and miR-199a-5p) was identified using random forest. Levels of miR-93-5p and miR-199a-5p were independently associated with the outcome, improving patient classification provided by the electronic health record. The experimentally validated targets of these miRNAs exhibited enrichment across diverse pathways, with telomere length quantification in an additional set of samples (n = 83) supporting the role of cell senescence in sequelae. Analysis of an external dataset refined the pathobiological fingerprint of pulmonary sequelae. Gene–drug interaction analysis revealed four FDA-approved drugs. Overall, this study advances our understanding of lung recovery in postacute respiratory infections, highlighting the potential of miRNAs and their targets for pulmoprotection.

## Graphical abstract

**Figure:**
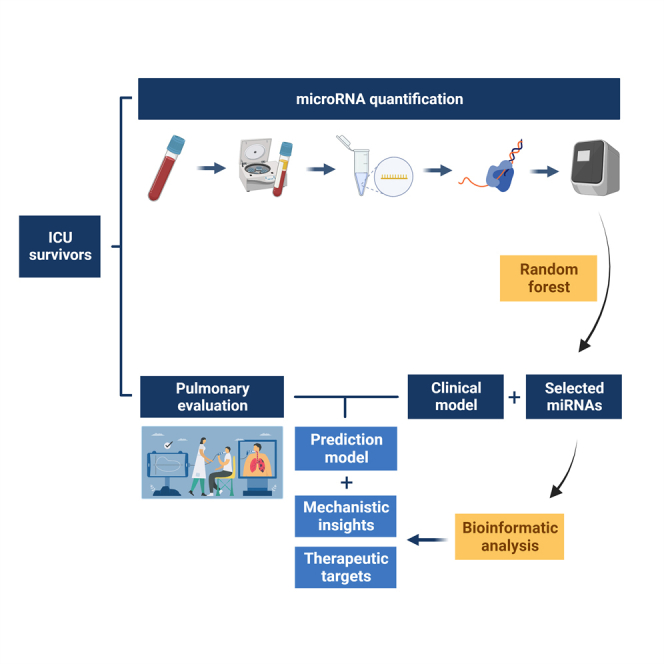

## Introduction

The evidence of postacute organ damage following severe acute respiratory syndrome-coronavirus-2 (SARS-CoV-2) infection especially applies to the pulmonary domain. Survivors of severe coronavirus disease 2019 (COVID-19) represent the group of patients at the highest risk of experiencing persistent respiratory abnormalities,^1^ with lung-diffusing capacity for carbon monoxide (D_LCO_) being the most affected parameter.^1^^,^^2^ Despite the efforts made since the onset of the COVID-19 pandemic, the research community underscores the urgent need to elucidate the pathobiological mechanisms underpinning COVID-19 sequelae. This step is crucial for developing early therapeutics and identifying patients with a heightened susceptibility to poor recovery.^3^

In the last decade, microRNAs (miRNAs), a class of evolutionarily conserved small noncoding RNAs (ncRNAs), have emerged as promising theranostic agents (i.e., combination of biomarkers and therapeutics enabling the diagnosis and treatment of medical conditions). miRNAs play pivotal roles as key regulators of gene expression networks, exerting a substantial impact on almost all biological processes. The development of miRNA-based therapeutic approaches is an active and promising field of research.^4^ The presence of miRNAs in the cell-free compartment, acting as mediators of cell-to-cell communication,^5^ has prompted their incorporation into molecular phenotyping. Circulating miRNAs hold a significant potential of translation as minimally invasive and cost-effective biomarkers.^6^ In this study, we aimed to assess, for the first time, the utility of miRNAs as theranostic agents for pulmoprotection in survivors of critical illness.

## Results

The study sample comprised 172 critical COVID-19 survivors admitted to 22 Spanish intensive care units (ICUs) between April 2020 and May 2021. All of the participants completed a follow-up visit within 1 year after hospital discharge (median [P25–P75] follow-up = 4.7 [2.78–7.33] months). The baseline and follow-up characteristics are shown in Table 1. Survivors exhibiting lung-diffusion impairment after hospital discharge (59.3% of patients, median D_LCO_ = 63.0 versus 87.5%) were older (median age = 62 versus 59 years), had a longer hospital (median = 40.0 versus 22.5 days) and ICU stay (median = 16 versus 12 days), and, among patients requiring invasive mechanical ventilation (IMV), had a longer duration of IMV (median = 11 versus 8 days). No differences in the prevalence of chronic lung disease were observed.

**Table 1:** Characteristics of study sample

	All N = 172	D_LCO_ <80 N = 102	D_LCO_ ≥80 N = 70	p	N
Sociodemographic data					
Age, years	61.0 (54.0–68.2)	62.0 (58.0–69.0)	59.0 (49.5–67.8)	0.021	172
Sex, female (%)	39 (22.7)	27 (26.5)	12 (17.1)	0.211	172
Body mass index	29.0 (26.0–33.7)	28.1 (25.6–31.2)	31.0 (27.4–36.7)	0.007	146
Smoking history (%)				0.678	164
Nonsmoker	94 (57.3)	53 (55.2)	41 (60.3)		
Former	64 (39.0)	40 (41.7)	24 (35.3)		
Current	6 (3.66)	3 (3.12)	3 (4.41)		
Comorbidities (%)					
Obesity	65 (37.8)	31 (30.4)	34 (48.6)	0.024	172
Hypertension	87 (50.6)	57 (55.9)	30 (42.9)	0.128	172
Diabetes mellitus (type 1/2)	41 (23.8)	23 (22.5)	18 (25.7)	0.767	172
Chronic heart disease	23 (13.5)	18 (17.8)	5 (7.14)	0.074	171
Chronic renal disease	12 (6.98)	10 (9.80)	2 (2.86)	0.126	172
Chronic lung disease	24 (14.0)	14 (13.7)	10 (14.3)	1.000	172
Asthma	12 (6.98)	8 (7.84)	4 (5.71)	0.764	172
Hospitalization					
Hospital stay, days	32.0 (18.0–53.0)	40.0 (22.0–58.0)	22.5 (16.0–35.8)	<0.001	171
ICU stay, days	14.0 (8.00–36.5)	16.0 (8.25–43.8)	12.0 (7.25–21.5)	0.005	172
ICU admission					
APACHE score	12.0 (9.00–15.0)	12.5 (10.0–15.0)	10.0 (8.00–14.8)	0.053	142
SOFA score	5.00 (4.00–7.00)	5.00 (4.00–7.00)	4.00 (3.75–7.00)	0.397	143
pH	7.43 (7.37–7.46)	7.42 (7.38–7.46)	7.44 (7.38–7.47)	0.452	160
Partial pressure of oxygen, mm Hg	75.0 (63.0–96.0)	76.7 (61.5–98.3)	74.0 (64.0–94.0)	0.481	157
Partial pressure of carbon dioxide, mm Hg	37.0 (32.0–42.0)	38.0 (32.9–41.2)	36.0 (32.0–42.0)	0.601	158
Bicarbonate, mmol/L	24.0 (22.0–25.8)	24.0 (22.0–25.9)	23.5 (22.0–25.2)	0.712	160
Oxygen saturation, %	95.0 (92.0–97.0)	95.0 (92.2–97.0)	95.0 (91.5–97.0)	0.876	165
PaO_2_-to-FiO_2_ ratio	109 (83.8–149)	113 (87.1–156)	100 (80.3–143)	0.256	153
Hemoglobin, g/dL	13.6 (12.5–14.7)	13.4 (12.0–14.7)	13.7 (13.0–14.6)	0.440	170
White blood count, ×10^9^/L	8.60 (6.58–11.8)	9.05 (6.25–11.7)	8.35 (6.84–11.8)	0.773	172
Lymphocyte count, ×10^9^/L	0.70 (0.50–1.03)	0.70 (0.50–1.04)	0.70 (0.47–1.00)	0.843	168
Neutrophil count, ×10^9^/L	7.40 (5.30–10.2)	7.67 (5.24–10.1)	7.18 (5.49–10.5)	0.890	167
Monocyte count, ×10^9^/L	0.40 (0.22–0.56)	0.40 (0.21–0.59)	0.40 (0.24–0.55)	0.588	167
Platelet count, ×10^9^/L	238 (194–304)	236 (199–287)	245 (191–322)	0.530	172
d-Dimer, mg/L	805 (543–1,405)	833 (520–1,630)	790 (622–1,293)	0.819	163
C-reactive protein, mg/dL	105 (38.8–188)	97.2 (38.2–182)	110 (45.8–195)	0.492	170
Glucose, mg/dL	151 (118–187)	146 (118–190)	156 (123–183)	0.803	170
Bilirubin, mg/dL	0.51 (0.39–0.70)	0.50 (0.37–0.65)	0.60 (0.42–0.72)	0.029	151
Aspartate transaminase, U/L	42.0 (28.0–63.2)	36.0 (26.0–63.0)	45.0 (30.5–66.0)	0.255	152
Alanine aminotransferase, U/L	43.0 (26.0–63.0)	35.5 (24.2–59.0)	49.0 (30.0–69.5)	0.056	165
Urea, mg/dL	50.0 (37.0–64.3)	53.4 (36.0–71.2)	47.3 (38.0–60.0)	0.351	169
Creatinine, mg/dL	0.83 (0.70–1.03)	0.87 (0.71–1.09)	0.80 (0.70–0.93)	0.136	172
Lactate dehydrogenase, U/L	434 (338–640)	448 (343–668)	415 (317–578)	0.257	151
EGFR, mL/min/1.73 m^2^	95.9 (78.8–105)	94.8 (69.5–103)	101 (88.4–107)	0.011	172
Pharmacological treatment (%)					
Corticosteroids	166 (97.6)	99 (99.0)	67 (95.7)	0.307	170
Anticoagulant	167 (98.8)	100 (100)	67 (97.1)	0.165	169
Antibiotics	160 (94.1)	97 (96.0)	63 (91.3)	0.319	170
Hydroxychloroquine	14 (8.24)	12 (11.9)	2 (2.90)	0.071	170
Remdesivir	33 (19.4)	22 (21.8)	11 (15.9)	0.454	170
Tocilizumab	48 (28.2)	33 (32.7)	15 (21.7)	0.167	170
Convalescent plasma	26 (15.4)	13 (13.0)	13 (18.8)	0.414	169
Procedures					
High-flow nasal cannula (%)	138 (85.2)	80 (84.2)	58 (86.6)	0.848	162
NIMV (%)	55 (32.4)	35 (34.7)	20 (29.0)	0.543	170
IMV (%)	120 (70.6)	72 (71.3)	48 (69.6)	0.944	170
IMV, days	9.00 (0.00–25.5)	11.0 (0.00–36.0)	8.00 (0.00–15.0)	0.024	170
Prone position (%)	89 (52.4)	56 (55.4)	33 (47.8)	0.412	170
Complications (%)					
Infectious complication	85 (49.7)	55 (54.5)	30 (42.9)	0.182	171
Bacterial pneumonia	55 (32.2)	33 (32.7)	22 (31.4)	0.996	171
ARDS	151 (88.3)	93 (92.1)	58 (82.9)	0.109	171
Pulmonary thromboembolism	19 (11.5)	13 (13.1)	6 (9.09)	0.584	165
Bacteremia	45 (26.5)	30 (30.0)	15 (21.4)	0.285	170
Acute renal failure	46 (26.9)	32 (31.7)	14 (20.0)	0.129	171
Hepatic dysfunction	47 (27.5)	27 (26.7)	20 (28.6)	0.928	171
Hyperglycemia	134 (78.4)	80 (79.2)	54 (77.1)	0.894	171
Follow-up parameters					
D_LCO_	74.5 (60.0–85.2)	63.0 (52.0–70.0)	87.5 (83.0–97.0)	<0.001	172
FEV (%)	86.9 (16.2)	82.2 (16.0)	93.9 (14.0)	<0.001	166
FVC (%)	84.6 (15.3)	80.1 (14.4)	91.4 (14.1)	<0.001	167
Persistent infiltrates (%)	32 (30.5)	19 (30.6)	13 (30.2)	1.000	105
Diffuse interstitial lung disease (%)	4 (3.81)	4 (6.45)	0 (0.00)	0.143	105
Fibrous tracts (%)	26 (24.8)	20 (32.3)	6 (14.0)	0.057	105
Emphysema (%)	4 (3.81)	3 (4.84)	1 (2.33)	0.643	105

ARDS, acute respiratory distress syndrome; FEV, forced expiratory volume; FiO_2_, fraction of inspired oxygen; FVC, forced vital capacity; PaO_2_, partial pressure of oxygen; SOFA, Sequential Organ Failure Assessment.

**Figure 1:**
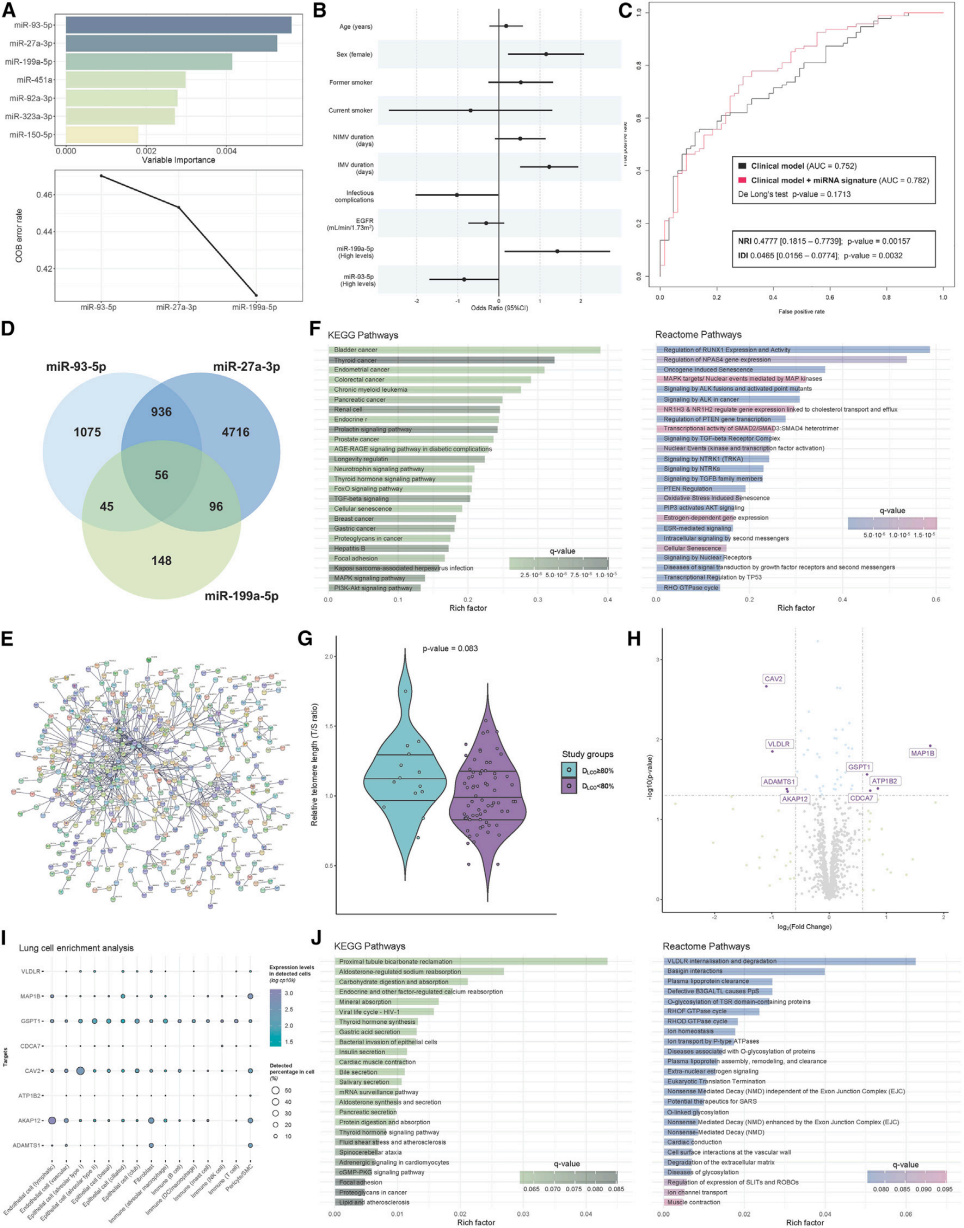
miRNAs as theranostic agents for pulmoprotection in postacute sequelae in survivors of critical illness (A) Random forest feature selection model. Top, the importance of the contribution of each miRNA to the model. Bottom, out-of-bag (OOB) error rate when including accumulated miRNAs in the model. The optimal model selection process does not include more miRNAs if they do not represent a significant improvement in the model prediction. The optimal model included 3 miRNAs. (B) Integration of selected miRNA into a clinical model for the prediction of lung-diffusion impairment (D_LCO_ <80%) previously constructed by our group.^2^ miRNA levels were dichotomized for the optimal cutoff. The final model presented was constructed using stepwise logistic regression. The odds ratio represents the risk change per 1 SD in continuous predictors. (C) Receiver operating characteristic (ROC) curves comparing the clinical model (black curve) with the clinical model incorporating the miRNA signature (red curve). The discriminative performance of both models is quantified by the area under the ROC curve (AUC). Reclassification analyses, the Integrated Discrimination Improvement (IDI), and continuous Net Reclassification Improvement (NRI) indexes were implemented to quantify the added value of miRNAs. (D) Venn diagram including the experimentally validated targetome of the selected miRNAs (TarBase version 8 database). (E) STRING protein–protein interaction network. The analysis included 1,133 genes (targets of at least 2 miRNAs). Edges indicate both physical and functional associations (interaction score cutoff is set at 0.95). (F) Top 25 terms (ranking based on q value and plotted according to the Rich Factor) of Kyoto Encyclopedia of Genes and Genomes (KEGG) and Reactome of the 1,133 target genes. The Rich Factor quantifies pathway enrichment, whereas the q value denotes significance. (G) Telomere length between D_LCO_ <80% and D_LCO_ ≥80% groups. (H) Volcano plot representing differential gene expression in an external whole-blood RNA-seq dataset (GSE228320) between D_LCO_ <80% and D_LCO_ ≥80% groups. Differential expression criteria were set at p < 0.05 and fold change ≥ 1.5. (I) Enrichment analysis of lung cell types based on single-cell RNA-seq data from the GTEx Project database. Each column shows a cell type and each row shows a gene. Point size indicates the number of cells where the gene was detected, and color represents expression level. (J) Top 25 enriched terms (ranked by q value) from KEGG and Reactome among the differentially expressed target genes. Functional enrichment analysis was performed using the clusterProfiler package (version 4.8.3) of the Bioconductor software (version 3.17). EGFR, estimated glomerular filtration rate; NIMV, noninvasive mechanic ventilation.

First, we constructed a miRNA-based prediction model of lung-diffusion impairment using a random forest feature selection procedure (Figure 1A). The optimal model included a 3-miRNA signature composed of miR-27a-3p, miR-93-5p, and miR-199a-5p. To explore the biomarker value of miRNA profiling, we used stepwise logistic regression models to integrate these miRNAs into a predictive clinical model of pulmonary dysfunction previously developed by our group.^2^ Demonstrating the potential of miRNAs as early biomarkers, the levels of miR-93-5p and miR-199a-5p measured upon ICU admission were not only independently associated with the outcome (Figure 1B) but also improved the patient classification provided by the electronic health record (Figure 1C).

Second, we used the acute 3-miRNA profile to elucidate the systemic mechanistic pathways relevant for designing pulmoprotective strategies. To accomplish this, we assessed experimentally validated miRNA–gene interactions (TarBase version 8).^7^ Among the 7,072 target genes, 1,133 were proved to be targets of at least two miRNAs (Figure 1D). As expected, the targets exhibited a high-confidence protein–protein interaction network (STRING) (Figure 1E).^8^ Functional analysis revealed an enrichment in multiple pathways that may anticipate poor resolution, including treatable traits previously described by our group and others such as fibrosis (in particular transforming growth factor-β signaling), metabolic alterations, tissue remodeling, and cell senescence (Figure 1F).^9^ To experimentally corroborate these findings, we quantified telomere length as a marker of cell senescence in an additional set of blood samples collected from survivors of critical COVID-19 after hospital discharge (n = 83).^10^ No significant differences were observed in potential confounding factors such as age, sex, and smoking history among survivors with and without posthospital discharge lung-diffusion impairment (Table 2). Consistent with the pathway analysis, we observed a trend toward shorter telomeres in survivors with diffusion impairment (Figure 1G).

**Table 2:** Characteristics of the study sample for telomere length substudy

	All N = 83	D_LCO_ <80 N = 69	D_LCO_ ≥80 N = 14	p	N
Age, years	60.0 (50.0–65.5)	61.0 (50.0–66.0)	57.0 (50.2–61.0)	0.284	83
Sex, female (%)	25 (30.1)	20 (29.0)	5 (35.7)	0.750	83
Smoking history (%)				0.868	79
Nonsmoker	28 (35.4)	22 (33.8)	6 (42.9)		
Former	48 (60.8)	40 (61.5)	8 (57.1)		
Current	3 (3.80)	3 (4.62)	0 (0.00)		

Perez-Pons and collaborators explored the pathophysiology of pulmonary damage caused by SARS-CoV-2 respiratory infection. The prognostic potential of circulating miRNAs provides valuable insights into postdischarge lung function impairment. The modulation of miRNAs and their targets emerges as an innovative strategy for early interventions in pulmonary protection.

To further delineate the mechanistic pathways associated with the 3-miRNA signature, we analyzed the levels of the 1,133 miRNA targets in an external RNA sequencing (RNA-seq) dataset (GEO: GSE228320). Survivors of critical COVID-19 with and without lung-diffusion impairment during follow-up (n = 37, median age = 60 years, females = 29.7%, median follow-up = 3.3 months) were strictly matched according to age, sex, ICU stay, and IMV duration. Eight transcripts (CAV2, MAP1B, VLDLR, GSPT1, ATP1B2, ADAMTS1, CDCA7, and AKAP12) exhibited differential expression among the study groups (Figure 1H). The analysis of an external single-cell RNA-seq dataset evidenced their expression in diverse lung cell subpopulations (Genotype-Tissue Expression Application Programming Interface [GTEx API] version 2) (Figure 1I).^11^ Functional analysis of the eight targets provided a more precise pathobiological fingerprint of pulmonary postacute sequelae, defining pathways implicated in viral and bacterial infection, protein glycosylation, extracellular matrix remodeling, cell migration, ion homeostasis, lipid and glucose metabolism, renal function, and cardiac alterations (Figure 1J). Four US Food and Drug Administration–approved drugs (acetyldigitoxin, deslanoside, digitoxin, and digoxin) that target ATP1B2 were retrieved from the gene–drug interaction analysis of the upregulated targets (Drug–Gene Interaction Database version 4.2.0).^12^

## Discussion

Collectively, these results contribute to a more refined understanding of the mechanistic pathways that underlie lung damage and recovery following acute respiratory infections. In this scenario, host-encoded miRNAs emerge as promising candidates for theranostic applications. The modulation of miRNA levels during the acute phase, and therefore, the subsequent targetome, constitute a new therapeutic approach for early pulmoprotection by preventing lung dysfunction and promoting repair. The use of small ncRNA therapeutics that regulate multiple underlying pathways may be especially profitable in a condition of a multifactorial nature such as postacute lung injury. Further evaluation of the miRNA targets in drug repositioning (ATP1B2) and other disease-modifying interventions (the use of senolytics to treat cell senescence) may also be worthwhile. In addition, circulating miRNAs demonstrate promising prognostic value as early biomarkers for predicting postdischarge lung function impairment. Complementary studies are necessary to confirm the findings, explore the role of miRNAs in prognostic and predictive enrichment, establish the pathogenetic link between molecular mechanisms and lung diffusion impairment, and extend the results to postinfectious disorders beyond COVID-19.

## Materials and methods

We conducted a multicenter study involving patients enrolled in the CIBERESUCICOVID project. This study was registered at ClinicalTrials.gov (NCT04457505). A detailed study protocol was previously published.^13^ Inclusion criteria encompassed individuals aged over 18 years, laboratory-confirmed SARS-CoV-2 infection, admission to the ICU, blood sample collection within the first 48 h of ICU admission, and pulmonary evaluation in a follow-up visit within the 12 months after hospital discharge. The outcome was the presence of lung-diffusion impairment during the follow-up (defined as a D_LCO_ <80%). miRNA quantification was performed as previously described.^14^ We quantified a 16-miRNA panel associated with the response to viral infection and/or end-organ damage, and experimentally linked to COVID-19 severity,^15^ using the gold standard technology qPCR in EDTA plasma samples. Relative quantification was performed using cel-miR-39-3p for normalization, and relative expression levels were log-transformed for statistical analysis. Genomic DNA (gDNA) for telomere measurements was isolated according to standard procedures from 50 μL blood using the DNeasy Blood & Tissue Kit (Qiagen) and stored at −20°C. DNA samples were diluted in 96-well plates to a fixed concentration of 10 ng/μL. A qPCR-based assay was performed to measure the relative telomere (T) length.^10^ The method compares mean T repeat sequence to a reference single copy (S) gene, for which the 36B4 gene was chosen, following the same principle as previously described,^16^ but with several optimizations of the experimental settings.^17^ DNA samples were amplified in a total reaction volume of 10 μL containing 1× iQ SYBR Green Supermix (BioRad), 2 μM ROX reference dye (Thermo Fisher) with either primer pairs for T or S amplification and 20 ng of DNA. For T repeat amplification, 300 nM of Human T FW primer and 900 nM of Human T RV primer were used, whereas for 36B4 amplification, the concentrations of primers were 300 nM of 36B4 FW and 500 nM of 36B4 RV. High-performance liquid chromatography–grade primers were used.

**Table:** 

Human T FW	5'-GGTTTTTGAGGGTGAGGGTGAG GGTGAGGGTGAGGGT-3'
Human T RV	5'-TCCCGACTATCCCTATCCCTA TCCCTATCCCTACCCTA-3'
Human 36B4 FW (S)	5'-CAGCAAGTGGGAAGGTGTAATCC-3'
Human 36B4 RV (S)	5'-CCCATTCTATCATCAACGGGTACAA-3'

All DNA samples were run on the qPCR (ABI Viia 7 Real-Time PCR system) as triplicates for both the T primer and 36B4 S primer, in a 384-well format. The thermal cycling profile was the same for both primers: 95°C incubation for 10 min followed by 35 cycles of 95°C for 15 s, 54°C for 2 min, and 72°C for 15 s. Interrun calibrators were included in each qPCR run comprising one gDNA sample with long T (human induced pluripotent stem cells) and another with short T (human umbilical vein endothelial cells, passage 5). A standard curve was generated from serially diluted reference DNA samples of human gDNA (Roche) to ensure primer and qPCR efficiency. The final relative T length (T/S ratio) for each sample was the ratio of T and S amplification, which was calculated based on the qPCR efficiency and relative to the interrun calibrators.^18^

The study was conducted in compliance with the Declaration of Helsinki and adhered to national and international data protection laws, receiving approval from the respective ethics committees.

## Data and code availability

The datasets that support the findings of this study are available from the corresponding author upon reasonable request.

## References

[1] Schlemmer F., Valentin S., Boyer L., Guillaumot A., Chabot F., Dupin C., Le Guen P., Lorillon G., Bergeron A., Basille D. (2023). Respiratory recovery trajectories after severe-to-critical COVID-19: a 1-year prospective multicentre study. Eur. Respir. J..

[2] González J., de Batlle J., Benítez I.D., Torres G., Santisteve S., Targa A.D.S., Gort-Paniello C., Moncusí-Moix A., Aguilà M., Seck F. (2023). Key Factors Associated With Pulmonary Sequelae in the Follow-Up of Critically Ill COVID-19 Patients. Arch. Bronconeumol..

[3] Saunders C., Sperling S., Bendstrup E. (2023). A new paradigm is needed to explain long COVID. Lancet Respir. Med..

[4] Khorkova O., Stahl J., Joji A., Volmar C.H., Wahlestedt C. (2023). Amplifying gene expression with RNA-targeted therapeutics. Nat. Rev. Drug Discov..

[5] Thomou T., Mori M.A., Dreyfuss J.M., Konishi M., Sakaguchi M., Wolfrum C., Rao T.N., Winnay J.N., Garcia-Martin R., Grinspoon S.K. (2017). Adipose-derived circulating miRNAs regulate gene expression in other tissues. Nature.

[6] Eckhardt C.M., Gambazza S., Bloomquist T.R., De Hoff P., Vuppala A., Vokonas P.S., Litonjua A.A., Sparrow D., Parvez F., Laurent L.C. (2023). Extracellular Vesicle-Encapsulated microRNAs as Novel Biomarkers of Lung Health. Am. J. Respir. Crit. Care Med..

[7] Karagkouni D., Paraskevopoulou M.D., Chatzopoulos S., Vlachos I.S., Tastsoglou S., Kanellos I., Papadimitriou D., Kavakiotis I., Maniou S., Skoufos G. (2018). DIANA-TarBase v8: a decade-long collection of experimentally supported miRNA–gene interactions. Nucleic Acids Res..

[8] Szklarczyk D., Gable A.L., Nastou K.C., Lyon D., Kirsch R., Pyysalo S., Doncheva N.T., Legeay M., Fang T., Bork P. (2021). The STRING database in 2021: customizable protein-protein networks, and functional characterization of user-uploaded gene/measurement sets. Nucleic Acids Res..

[9] García-Hidalgo M.C., Peláez R., González J., Santisteve S., Benítez I.D., Molinero M., Perez-Pons M., Belmonte T., Torres G., Moncusí-Moix A. (2022). Genome-wide transcriptional profiling of pulmonary functional sequelae in ARDS- secondary to SARS-CoV-2 infection. Biomed. Pharmacother..

[10] Chatterjee S., de Gonzalo-Calvo D., Derda A.A., Schimmel K., Sonnenschein K., Bavendiek U., Bauersachs J., Bär C., Thum T. (2018). Leukocyte telomere length correlates with hypertrophic cardiomyopathy severity. Sci. Rep..

[11] Lonsdale J., Thomas J., Salvatore M., Phillips R., Lo E., Shad S., Hasz R., Walters G., Garcia F., Young N. (2013). The Genotype-Tissue Expression (GTEx) project. Nat. Genet..

[12] Freshour S.L., Kiwala S., Cotto K.C., Coffman A.C., McMichael J.F., Song J.J., Griffith M., Griffith O.L., Wagner A.H. (2021). Integration of the Drug–Gene Interaction Database (DGIdb 4.0) with open crowdsource efforts. Nucleic Acids Res..

[13] Torres A., Motos A., Ceccato A., Bermejo-Martin J., de Gonzalo-Calvo D., Pérez R., Barroso M., Pascual I.Z., Gonzalez J., Fernández-Barat L. (2022). Methodology of a Large Multicenter Observational Study of Patients with COVID-19 in Spanish Intensive Care Units. Arch. Bronconeumol..

[14] de Gonzalo-Calvo D., Molinero M., Benítez I.D., Perez-Pons M., García-Mateo N., Ortega A., Postigo T., García-Hidalgo M.C., Belmonte T., Rodríguez-Muñoz C. (2023). A blood microRNA classifier for the prediction of ICU mortality in COVID-19 patients: a multicenter validation study. Respir. Res..

[15] de Gonzalo-Calvo D., Benítez I.D., Pinilla L., Carratalá A., Moncusí-Moix A., Gort-Paniello C., Molinero M., González J., Torres G., Bernal M. (2021). Circulating microRNA profiles predict the severity of COVID-19 in hospitalized patients. Transl. Res..

[16] Cawthon R.M. (2002). Telomere measurement by quantitative PCR. Nucleic Acids Res..

[17] Martinez-Delgado B., Yanowsky K., Inglada-Perez L., Domingo S., Urioste M., Osorio A., Benitez J. (2011). Genetic anticipation is associated with telomere shortening in hereditary breast cancer. PLoS Genet..

[18] Willeit P., Willeit J., Mayr A., Weger S., Oberhollenzer F., Brandstätter A., Kronenberg F., Kiechl S. (2010). Telomere length and risk of incident cancer and cancer mortality. JAMA.

